# Optimal dose of perineural dexmedetomidine for interscalene brachial plexus block to control postoperative pain in patients undergoing arthroscopic shoulder surgery: A prospective, double-blind, randomized controlled study

**DOI:** 10.1097/MD.0000000000010440

**Published:** 2018-04-20

**Authors:** Hong Soo Jung, Kwon Hui Seo, Jae Hyuk Kang, Jin-Young Jeong, Yong-Shin Kim, Na-Re Han

**Affiliations:** a Department of Anesthesiology and Pain Medicine, St. Vincent's Hospital, College of Medicine, The Catholic University of Korea; b Department of Orthopedic Surgery, St. Vincent's Hospital, College of Medicine, The Catholic University of Korea, Jungbu-daero, Paldal-gu, Suwon-si, Gyeonggi-do, Republic of Korea.

**Keywords:** arthroscopy, brachial plexus block, dexmedetomidine, postoperative pain, ropivacaine, shoulder

## Abstract

**Background:**

Adjuvant perineural dexmedetomidine can be used to prolong the analgesic effect of interscalene brachial plexus block (ISB). We investigated the optimal dose of dexmedetomidine in ISB for postoperative analgesia in patients undergoing arthroscopic shoulder surgery.

**Methods:**

One hundred patients scheduled for elective shoulder arthroscopic surgery were enrolled in this randomized, double-blind study. Ultrasound-guided ISB was performed before general anesthesia using 22 mL of ropivacaine 0.5% combined with 1, 1.5, or 2 μg/kg of dexmedetomidine (group D1, D2, and D3, respectively) or with normal saline as a control (group R, n = 25 per group). The primary outcome was the duration of analgesia (DOA), numeric pain rating scale (NRS), and consumption of additional analgesics during 36 h after ISB. Secondary outcome included durations of motor and sensory block (DOM and DOS), hemodynamic variables and sedation and dyspnea scores.

**Results:**

Ninety-seven patients completed the study. The DOS, DOM, and DOA were significantly longer in the dexmedetomidine groups than in group R. The DOA was significantly longer in group D3 than in groups D1 (*P* = .026) and D2 (*P* = .039). The DOA was 808.13 ± 179.97, 1032.60 ± 288.14, 1042.04 ± 188.13, and 1223.96 ± 238.06 min in groups R, D1, D2, and D3, respectively. The NRS score was significantly higher in group R than in the dexmedetomidine groups 12 h after ISB (*P* < .001) and significantly lower in group D3 than in the other groups 18 h after ISB (*P* = .02). The incidence of hypotension was higher in groups D2 and D3 than in group R during surgery (*P* = .008 and *P* = .011, respectively). There were no significant differences in consumption of rescue analgesics, sedation, and dyspnea scores between the study groups.

**Conclusion:**

Perineural dexmedetomidine 2 μg/kg could be the optimal dose in ISB for arthroscopic shoulder surgery in that it provides an adequate DOA. However, this dose was associated with increased risk of hypotension.

## 1 Introduction

Postoperative pain after arthroscopic shoulder surgery is severe, with a reported numeric pain rating scale (NRS) of 4 to 5.^[1,2]^ The pain on the night following surgery may be very severe and disturbs sleep, but access to care during these hours is often limited.^[3]^ Use of systemic analgesics to relieve this nocturnal pain can have adverse effects, including nausea, vomiting, respiratory depression, and sleep disturbance.^[4]^ Several methods have been tested for postoperative pain control in these patients, including intra-articular injection, single-shot interscalene brachial plexus block (ISB), and continuous ISB. Single-shot ISB is less invasive, technically easier to perform, and has fewer complications than the other methods, therefore, is widely used.^[5]^ However, single-shot ISB has a limited duration of action, cannot control pain during the night. Ropivacaine, a long-acting local anesthetic agent, is usually used for prolongation of analgesia, but the average duration of analgesia (DOA) is only about 8 to 14 h, which is not enough to control postoperative pain through the night.^[6]^

Various adjuvants have been used to prolong the duration of nerve blocks, including epinephrine, clonidine, and dexmedetomidine.^[7]^ Dexmedetomidine is a highly selective α_2_ adrenergic receptor agonist, and can be expected to have a longer DOA than other adjuvants without neurotoxicity.^[7]^ Several animal experiments have demonstrated that dexmedetomidine prolongs the effect of a nerve block,^[8,9]^ and clinical trials have also confirmed the favorable effect of dexmedetomidine on peripheral nerve and brachial plexus blocks.^[10–12]^ However, dexmedetomidine has been used as wide range of doses (20–150 μg) and there are no relevant published dosing guidelines or recommendations.^[11,13,14]^ A few studies have compared the prolongation effect of different doses of perineural dexmedetomidine in nerve block, but the appropriate dose in ISB for postoperative analgesia and preventing adverse effects in patients undergoing arthroscopic shoulder surgery is not yet known.

The aim of this study was to compare the effects of different doses of perineural dexmedetomidine with ropivacaine on the perioperative pharmacodynamic parameters of ultrasound-guided ISB and to investigate the optimal dose in patients undergoing arthroscopic shoulder surgery.

## 2 Materials and methods

The study was approved by the Catholic University, St. Vincent Hospital Institutional Review Board (approval number: VC15MISI0145) and is listed on a registry recognized by the World Health Organization (https://cris.nih.go.kr, KCT0001848). Written informed consent was obtained from all patients.

### 2.1 Patient population

We recruited 113 patients (aged 30–80 years) who were scheduled for elective arthroscopic shoulder surgery under general anesthesia in combination with ISB from August 19, 2015 to April 4, 2016. This trial was performed at the Catholic University, St. Vincent Hospital, which is a secondary university hospital located in a medium-sized city of South Korea. The exclusion criteria were as follows: American Society of Anesthesiologists (ASA) physical status of III or higher, body mass index > 35 kg/m^2^, pregnancy, coagulopathy, bleeding diathesis, a significant psychiatric or cognitive condition interfering with the ability to provide consent or assessment, uncontrolled respiratory disease, significant cardiovascular disease (second-degree or third-degree heart block, congestive heart failure, symptomatic coronary artery disease), preexisting neurologic deficits or neuropathy affecting the brachial plexus, hypersensitivity or allergy to dexmedetomidine or ropivacaine, and chronic opioid use (>6 months).

### 2.2 Study protocol

The enrolled patients were randomly assigned to 1 of 4 groups using a computer-generated randomization table with an allocation ratio of 1:1:1:1. The randomization scheme was generated using the website http://www.randomization.com. The randomization was performed by a resident anesthesiologist who was not involved in the anesthetic management of the patients or the collection of data. The patients, an anesthesiologist performing the ISB, and the nurses and anesthesiologists who measured the study outcome parameters were all blinded to study group allocation.

All study participants received a perineural solution according to their group allocation as follows: 20 mL of 0.5% ropivacaine with normal saline 2 mL (group R, controls); 20 mL of 0.5% ropivacaine with 2 mL of dexmedetomidine 1 μg/kg (group D1); 20 mL of 0.5% ropivacaine with 2 mL of dexmedetomidine 1.5 μg/kg (group D2); and 20 mL of 0.5% ropivacaine with 2 mL of dexmedetomidine 2 μg/kg (group D3). The dose of dexmedetomidine was determined according to each patient's ideal body weight (IBW), which was calculated using Broca formula: IBW (kg) = Height (cm) − x, where x is 100 for men and 105 for women. The study drugs were ropivacaine (150 mg/20 mL ropivacaine hydrochloride; Naropin, AstraZeneca LP, Wilmington, DE) and dexmedetomidine (100 μg/mL dexmedetomidine hydrochloride; Precedex, Hospira Inc, Lake Forest, IL). The drug solutions were prepared by nurses not involved in the study.

Electrocardiography, peripheral oxygen saturation (SpO_2_), noninvasive blood pressure (BP), and bispectral index (BIS) monitoring were started on arrival in the operating room. After sterile skin preparation with chlorhexidine and skin infiltration with 1% lidocaine, a high-frequency linear array transducer (6–3 MHz; SonoSite S-nerve, Inc., Bothell, WA) probe protected by a sterile dressing was placed in the transverse plane over the interscalene groove to visualize the fifth cervical spinal nerve (C5) roots of the brachial plexus between the anterior and middle scalene muscles. A 50-mm 22-gauge needle was then inserted in plane with the ultrasound probe until the needle tip was adjacent to the C5 root. The study solution was injected circumferentially around the C5 nerve root. A nerve stimulator was not used. ISB was performed by a single trained anesthesiologist who was blinded to group allocation. All operations were performed by a single experienced shoulder surgeon.

### 2.3 Measurements

BP, heart rate (HR), and SpO_2_ were recorded 1 and 5 min after ISB. Thereafter, general anesthesia was induced with intravenous (IV) propofol 2 to 2.5 mg/kg and rocuronium 0.6 mg/kg. After endotracheal intubation, anesthesia was maintained with a mixture of 50% nitrous oxide and 4% to 7% desflurane in oxygen. The concentration of desflurane was regulated to maintain a BIS of 40 to 60. After induction of general anesthesia, the patient's position was changed to lateral decubitus. BP, HR, and SpO_2_ were recorded at the start of the operation, 30 and 60 min after the start of the operation. Ephedrine 10 mg was administered intraoperatively when hypotension occurred; hypotension was defined as a 30% decrease in mean BP from the baseline value. Atropine 0.5 mg was administered when bradycardia occurred; bradycardia was defined as an HR < 45 beats/min. All patients were administered IV ramosetron 0.3 mg at the end of skin closure and IV metoclopramide 10 mg was administered in patients who experienced postoperative nausea and vomiting. Sugammadex 200 mg was administered for reversal of rocuronium at the end of the operation.

All patients were transferred to the recovery room after emergence from general anesthesia. BP, HR, and SpO_2_ were monitored and the sedation score, motor power, sensory function, and degree of dyspnea were checked during recovery period. For pain assessment, the patients were asked to rate their pain at rest on a 0 to 10 NRS (0, no pain; 10, worst imaginable pain). Sedation was evaluated using the Richmond Agitation-Sedation Scale (RASS) (−5, unarousable; −4, deep sedation; −3, moderate sedation; −2, light sedation; −1, drowsy; 0, alert and calm; 1, restless; 2, agitated; 3, very agitated; 4, combative).^[15]^ A modified Bromage scale was used to assess motor power in the upper limbs (0, able to raise the extended arm to 90° for 2 s; 1, able to flex the elbow and move the fingers but unable to raise the extended arm; 2, unable to flex the elbow but able to move the fingers; and 3, unable to move the arm, elbow, or fingers).^[16]^ Sensory function was tested by an alcohol swab and pinprick in the C5 dermatomes of the brachial plexus. The degree of dyspnea was assessed using the revised Borg scale for grading severity of dyspnea (0, nothing at all; 1, just noticeable; 2, very slight; 3, slight; 4, slight-moderate; 5, moderate; 6, some difficulty; 7, moderately severe; 8, severe; 9, very severe; 10, panic level, maximal shortness of breath) and graded in 4 stages (0, none; 1–3, mild; 4–6, moderate; ≥7, severe).^[17]^ The numbers of patients who complained of dyspnea (mild or worse) and hypoxemia (SpO_2_ < 93%) were recorded.

Before discharge from the recovery room, IV patient-controlled analgesia (PCA, fentanyl 0.1–0.2 μg/kg per mL, ketorolac 0.01–0.02 mg/kg per mL, ramosetron 0.3 mg) contained in a PCA device (Accufuser, Ace Medical, Seoul, Korea) was connected (total volume 100 mL, rate 2 mL/h, bolus 1 mL and lockout time 15 min) but not opened.

### 2.4 Data collection

After discharge from the recovery room, BP, HR, SpO_2_, NRS score, sedation score, dyspnea scale, and motor power were evaluated at 6, 12, 18, 24, and 36 h after ISB by the study nurse. The durations of sensory block (DOS), motor block (DOM), and analgesia, along with the amount of rescue analgesics used were also recorded. All patients were provided with a postoperative questionnaire to document the time when they regained normal or presurgical strength in their shoulder and elbow; when numbness, tingling, weakness, and anything that is not normal related to the nerve block wore off. The DOS was defined as the time from ISB to complete recovery of normal sensation (full waning of block). The DOM was defined as the time interval between ISB and complete recovery of motor power in the C5-Thoracic1 dermatomes. Motor power was evaluated in the fingers, elbow, and arm using the modified Bromage scale. The DOA was defined as the time interval between ISB and the first need for rescue analgesics. The PCA device was opened when sensation in the shoulder started to be recovered, and the patients were educated to push the button when pain occurred. We defined DOA as the time until first push of the PCA button after ISB. Nurses caring for the patient were instructed to note the time in which the patient push the PCA button for the first time, thereafter, assessed NRS every 30 min. When a patient reported a resting NRS pain score ≥4 even after pushing the PCA button, IV tramadol 50 mg was injected. If the pain (NRS score ≥4) continued after administration of tramadol, IV diclofenac 75 mg was injected.

### 2.5 Statistical analysis

The sample size was calculated based on the assumption that dexmedetomidine would prolong the DOA by 20%. We used the study data published by Esmaoglu et al, who showed that perineural dexmedetomidine prolonged the DOA from 887 ± 261 to 1009 ± 164 min in patients who had received an axillary brachial plexus block.^[10]^ We assumed mean DOA of 890 min, a standard deviation of 170 min, and that a 20% prolongation of analgesia would be statistically significant. This calculation established that a sample size of 22 was required in each group for a type I error of 0.05 and a type II error of 0.2. Allowing for a drop-out rate 15%, a total of 100 patients were required.

The statistical analysis was performed using SPSS for Windows, version 20.0 software (IBM Corp., Armonk, NY) and R 3.2.4 (R Foundation for Statistical Computing, Vienna, Austria). Numeric variables are presented as the mean and standard deviation and categorical data are presented using counts and percentages. Continuous variables were tested for normal distribution using the Kolmogorov–Smirnoff test. For normally distributed data, the statistical analysis was carried out by 1-way analysis of variance followed by Bonferroni or Tukey post hoc test. For non-normally distributed data, the statistical analyses were performed using the Kruskal–Wallis test and the Mann–Whitney test followed by Bonferroni correction of the significance level. The incidences of the categorical data were analyzed using the chi-squared test with Sheff test for post hoc comparison. Kaplan–Meier survival curves were constructed to compare time to first request for rescue analgesics (when pushing the PCA button) between the groups. A *P* value < .05 was considered to be statistically significant.

## 3 Results

### 3.1 Patients

**Figure 1:**
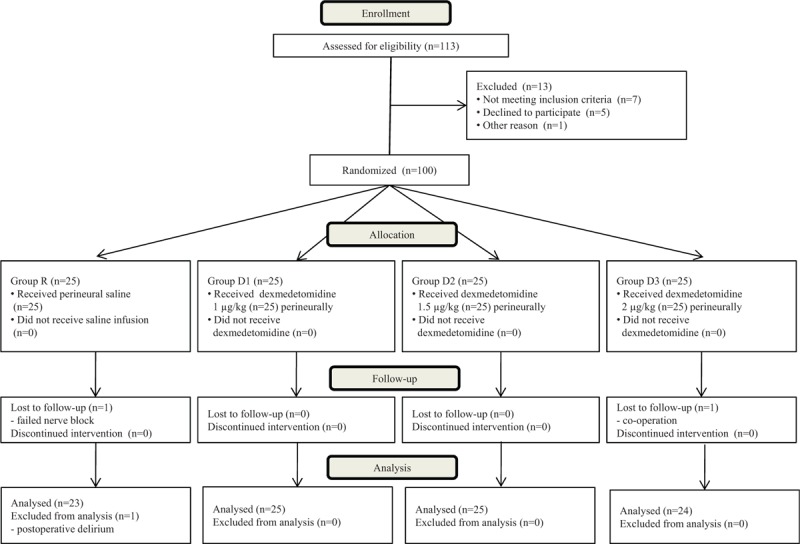
Consort flow diagram.

One hundred patients were enrolled in the study. In group R, data were excluded for 1 patient because of incomplete block and for another with postoperative delirium, and 1 patient in group D3 was excluded because carpal tunnel release was performed on the contralateral hand, leaving data for 97 patients available for analysis (23 in group R, 25 in group D1, 25 in group D2, and 24 in group D3; Fig. 1).

The patient characteristics and perioperative data are shown in Table 1. There were no statistically significant differences in the patient characteristics in the 4 groups, except that the patient weights in groups D1 and D3 were significantly higher than those in group R (*P* = .049 and *P* = .026, respectively) and the proportion of patients with ASA physical status I was significantly higher in group R than in groups D1 and D3 (*P* = .045 and *P* = .012, respectively).

**Table 1:**
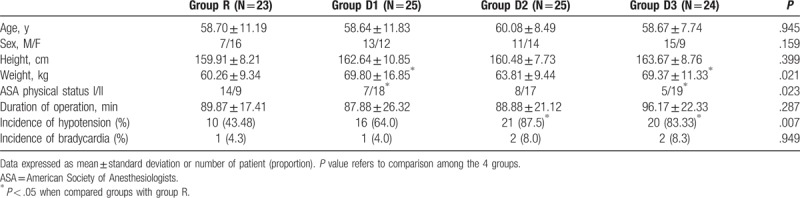
Patient characteristics and perioperative data.

### 3.2 Hemodynamic variables

**Figure 2:**
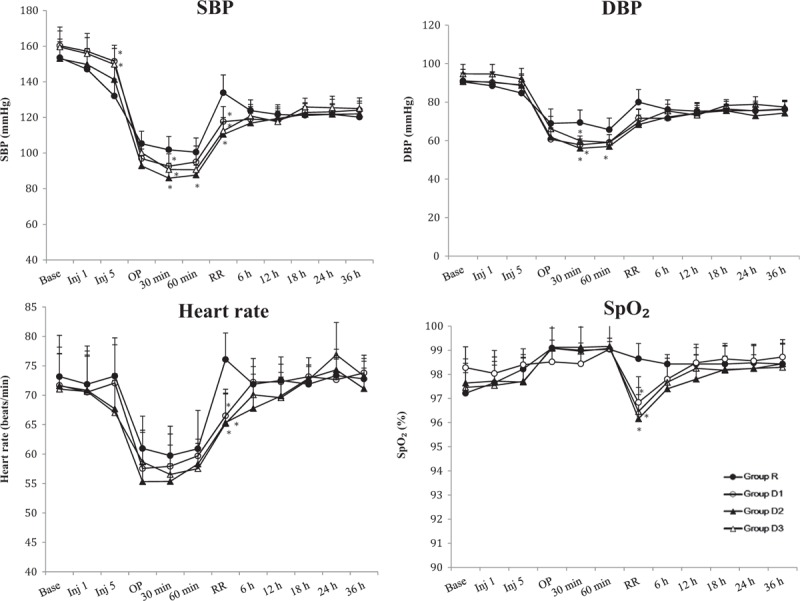
Systolic and diastolic blood pressure (SBP and DBP), heart rate, and peripheral oxygen saturation (SpO_2_) during study period in the 4 groups. ^∗^*P* < .05 versus group R. Base: before interscalene block (ISB), Inj 1: 1 min after ISB, Inj 5: 5 min after ISB, OP: at the start of the operation, 30 min: 30 min after the start of the operation, 60 min: 60 min after the start of the operation, RR: in the recovery room, 6 h: 6 h after ISB, 12 h: 12 h after ISB, 18 h: 18 h after ISB, 24 h: 24 h after ISB, 36 h: 36 h after ISB.

As shown in Fig. 2, there was no difference in the hemodynamic variables between the groups at baseline. The hemodynamic changes were very similar in the 4 groups, BP and HR decreased during operation but normalized in the recovery room. Comparing the 4 groups, there were a few statistically significant differences in the hemodynamic variables intraoperatively. There were significant differences in systolic BP 5 min after ISB, 30 and 60 min after the start of the operation and in the recovery room between the 4 groups. Systolic BP was significantly higher in groups D1 and D3 than in group R at 5 min after ISB (*P* = .004 and *P* = .012, respectively). Thirty minutes after the start of surgery, systolic BP was significantly lower in the dexmedetomidine groups than in group R (*P* = .04 for group D1, *P* < .001 for group D2 and *P* = .008 for group D3). In group D2, systolic BP was significantly lower than in group R at 60 min after the start of surgery (*P* = 0.01). In the recovery room, systolic BP was significantly lower in the dexmedetomidine groups than in group R (*P* = .007 for group D1, *P* < .001 for groups D2 and D3). The diastolic BP was significantly lower in the dexmedetomidine groups than in group R at 30 min after the start of surgery (*P* < .001 for groups D1 and D2, *P* = .001 for group D3). Sixty minutes after the start of surgery, diastolic BP was significantly lower in group D2 than in group R (*P* = .006). HR and SpO_2_ were significantly lower in the dexmedetomidine groups when compared with the values in group R in the recovery room (*P* = .016, .005, and .004 for HR and *P* = .036, .001 and .006 for SpO_2_ in groups D1, D2, and D3, respectively). There was no significant difference in hemodynamic variables between the dexmedetomidine groups.

The incidence of hypotension was significantly greater in groups D2 and D3 than in group R (*P* = .008 and .011 for group D2 and D3, respectively, Table 1) during the perioperative period. The incidence of bradycardia during the perioperative period was comparable between the 4 groups (Table 1).

### 3.3 Postoperative side effects in the recovery room

There was no significant difference in sedation score in the recovery room (Table 2). One patient in group D3 checked an RASS score of −3 (moderate sedation) that changed to 0 (alert) at 4 h after discharge from the recovery room. All other patients checked RASS scores of −2 (light sedation) to 0 in the recovery room.

**Table 2:**
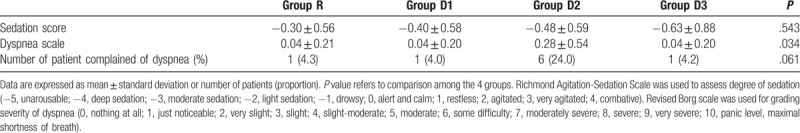
Sedation score and incidence of dyspnea in the recovery room in 4 groups.

The Kruskal–Wallis test revealed a significant difference in the dyspnea scale between the 4 groups that disappeared when Bonferroni post hoc test was used to compare the groups (Table 2). The incidence of dyspnea was highest in group D2 but there was no significant difference between 4 groups (Table 2). One patient in group D2 developed moderate dyspnea that resolved 18 h after ISB. Other patients who complained of dyspnea had mild symptoms, regardless of group allocation.

One, two, and two patients in groups D1, D2, and D3, respectively, developed hypoxemia, which recovered to the baseline SpO_2_ value in all cases within 6 h of ISB with oxygen 5 L/min via face mask. No patient required any invasive airway or mask ventilation after emergence from general anesthesia.

### 3.4 Duration of nerve block and pain assessment

The modified Bromage scale score was used to assess motor nerve block, and was found to be significantly higher in groups D1 and D3 than in group R 12 h after ISB (*P* = .003 for group D1 and *P* < .001 for group D3, Table 3). Eighteen hours after ISB, the modified Bromage scale score was significantly higher in group D3 than in group R (*P* = .003, Table 3). There were no significant differences in motor nerve block between the groups at the other time points.

**Table 3:**
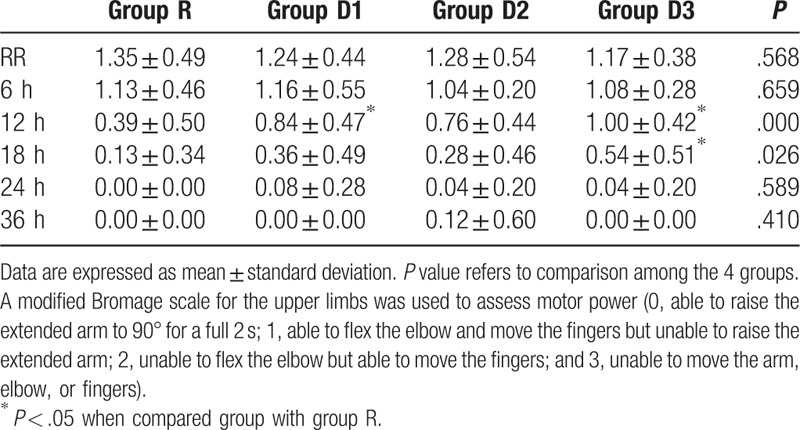
Motor block assessed by modified Bromage scale.

**Figure 3:**
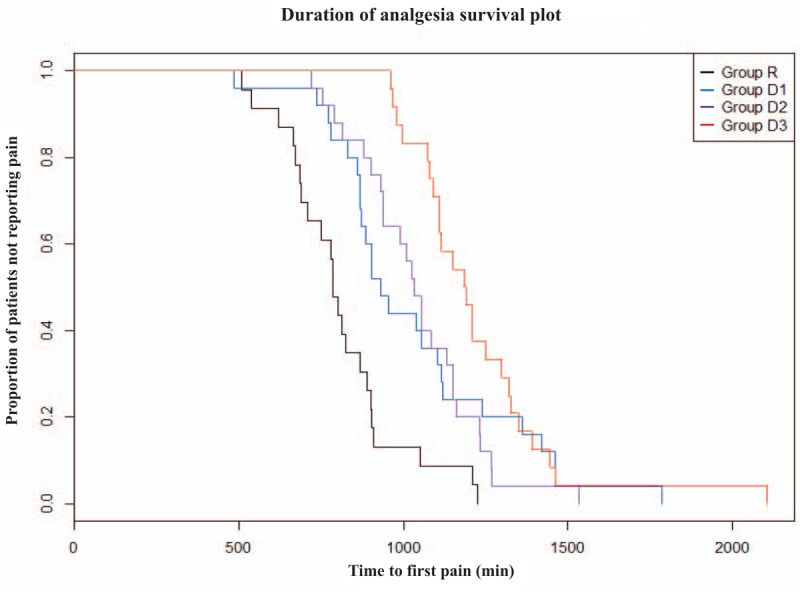
Kaplan–Meier curve for duration of analgesia. Pooled log-rank test *P* < .001.

The DOS, DOM, and DOA were significantly longer in the dexmedetomidine groups than in group R (Table 4). When the dexmedetomidine groups were compared, group D3 was associated with the longest DOS, DOM, and DOA (Table 4, Fig. 3), but only DOA was significantly different from that in groups D1 and D2. There were no significant differences in DOS, DOM, and DOA between groups D1 and D2.

**Table 4:**
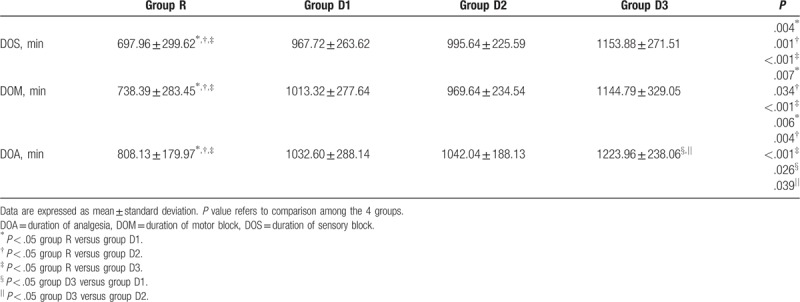
Duration of sensory, motor block, and analgesia in 4 groups.

**Figure 4:**
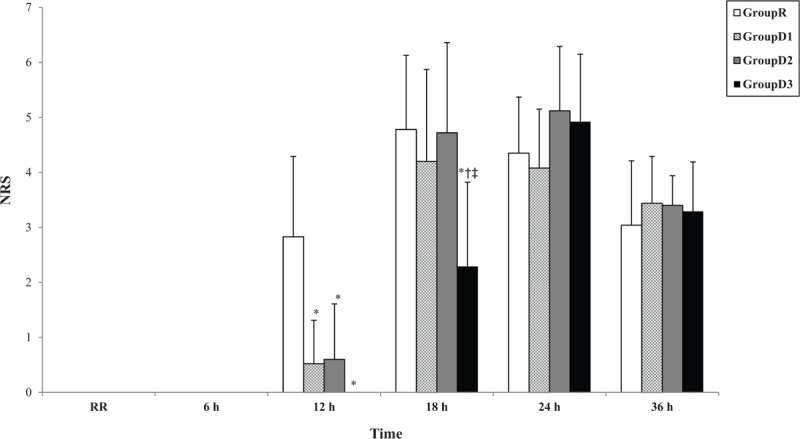
Numeric pain rating scale time courses. ^∗^*P* < .05 when compared group with group R; ^†^*P* < .05 when compared group with group D1; ^‡^*P* < .05 when compared group with group D2. Time values indicated by RR through 36 h are as in Fig. 2.

As shown in Fig. 4, the NRS score was significantly lower in the dexmedetomidine groups than in group R 12 h after ISB (*P* < .001 for groups D1, D2, and D3) and was also significantly lower in group D3 than in the other groups 18 h after ISB (*P* = .002, .042, and .013 when compared with groups R, D1, and D2, respectively). At 18 h after ISB, group D3 reported more than 2-point difference in NRS scores when compared with the other groups. There was no significant difference in pain scores assessed 24 and 36 h after ISB in any of the groups. There were no significant differences in the total dose of rescue analgesics or number of patients in whom rescue analgesics were administered between any of the groups for 36 h (Table 5).

**Table 5:**
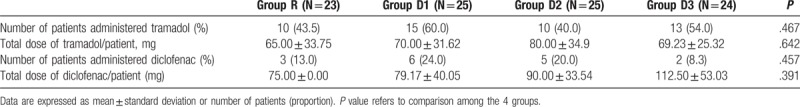
Supplementary intravenous analgesia in 4 groups.

We did not encounter any ISB-related complications, such as neuropraxia, vascular puncture, or hypersensitivity reaction to local anesthetics.

## 4 Discussion

The aim of the present study was to determine the optimal dose of perineural dexmedetomidine for ISB in patients undergoing arthroscopic shoulder surgery. We found that 1 μg/kg or more of perineural dexmedetomidine was associated with prolongation of the DOM, DOS, and DOA, but could not reduce the postoperative analgesic requirement. Perineural dexmedetomidine dose of 2 μg/kg could prolong the DOA for about 20 h. Transient hypotensive episodes were more common in patients who received perineural dexmedetomidine at a dose of 1.5 and 2 μg/kg but there were no serious complications.

Arthroscopic shoulder surgery can cause severe pain, especially during the first 24 postoperative hours.^[1]^ Cho et al reported that resting pain in patients undergoing arthroscopic rotator cuff repair was very severe, with visual analog scale scores of 7 to 9 immediately after surgery, 5 at 24 h postoperatively, and 4 to 5 for 84 h postoperatively despite administration of IV PCA or an infusion of local anesthetics in the subacromial space.^[2]^ Even with a long-acting local anesthetic like ropivacaine, if ISB is performed during the daytime, patients would report their first episodes of severe pain during the night because of the limited DOA. Nighttime pain can cause sleep disturbance and lead to a vicious cycle of acute pain.^[3]^ Continuous ISB has been recommended to overcome the limited DOA of a single-shot injection and to reduce opioid consumption. However, the results are affected by the catheter fixation technique because of the mobile nature of the surrounding area,^[18]^ and complications such as mechanical displacement of catheters, leaking, and consequent failure can occur. Importantly, despite the profound analgesia afforded by continuous ISB, supplemental analgesics are still required.^[5]^

Many studies have attempted to extend the duration of single injection peripheral nerve or brachial plexus block. The ideal adjuvant to local anesthetics would have the effect of prolonging the DOA without a substantial risk of neurotoxicity. Dexmedetomidine is now one of the most preferable adjuvants used in nerve block.^[11]^ In 2008, Brummett et al were the first to report that dexmedetomidine improved the duration of sciatic nerve block without inducing neurotoxicity in rats.^[8]^ Several clinical trials have investigated the beneficial effect of perineural dexmedetomidine on DOA, but mainly evaluated the effect of single doses.^[10,11,13,19]^ Most studies of the effect of perineural dexmedetomidine in brachial plexus block have been performed using a dose of 1 μg/kg.^[20–23]^ These studies used different local anesthetics in various concentrations and doses, and the DOA with dexmedetomidine was in the range of 6 to 8 h^[20,22,23]^ to 12 to 13 h.^[21]^ Higher doses of dexmedetomidine (100 or 150 μg) as an adjuvant to axillary or ISB extended the DOA or DOS to 16 to 18 h.^[10,13]^

An experimental study in a rodent model initially confirmed that dexmedetomidine could increase the DOS in a dose-dependent fashion when added to ropivacaine for sciatic nerve block.^[9]^ That study also demonstrated that extremely high doses of dexmedetomidine (20 μg/kg) did not lead to neurotoxicity. A few clinical studies have compared different doses of perineural dexmedetomidine.^[14,24,25]^ Zhang et al^[14]^ evaluated the effects of dexmedetomidine 50 and 100 μg with 40 mL of 0.33% ropivacaine in axillary brachial plexus block using a nerve stimulator. The DOS was significantly prolonged by adding 100 μg but not 50 μg of dexmedetomidine in their study. A volunteer study compared the pharmacodynamic data for placebo and dexmedetomidine 50, 100, or 150 μg mixed with 3 mL of 0.75% ropivacaine in ulnar nerve block and showed that the DOS increased to 8.7, 16.4, 20.4, and 21.2 h, respectively.^[25]^ Two subjects in the group who received 150 μg of dexmedetomidine developed postblock paresthesia for 72 h, so the investigators concluded that 100 μg of dexmedetomidine represents a balance between efficacy and side effects. However, there were only 6 cases in each group and surgical stimulation was excluded, so it is difficult to extrapolate those results to the clinical setting. As shown in previous studies, perineural dexmedetomidine can have peripheral and systemic effects in a dose-dependent fashion. However, most studies used fixed doses of dexmedetomidine. We determined the comparative doses of dexmedetomidine as 1, 1.5, and 2 μg/kg because side effects can occur with systemic absorption. Indeed, dexmedetomidine is well absorbed systemically after extravascular injection^[26]^ and produces similar hemodynamic changes with IV infusions.^[27]^ Most of the studies have demonstrated that 1 μg/kg of perineural dexmedetomidine significantly prolongs the DOA in various settings, so we chose the minimal dose as 1 μg/kg.

The results of the present study are in line with those of other studies comparing different doses of perineural dexmedetomidine. Increasing the dose of dexmedetomidine prolonged the DOS and DOA. However, there was no statistically significant difference in DOA between the group treated with perineural dexmedetomidine 1 μg/kg and the group treated with 1.5 μg/kg. Only patients treated with dexmedetomidine 2 μg/kg showed a significantly prolonged DOA that could control night-time pain; the DOA at this dose was about 3 h longer than that in patients treated with the other doses of dexmedetomidine and about 7 h longer than that in the placebo group. In respect of DOM, there was no potentiating effect of increased doses of dexmedetomidine. This finding is similar to that of another dose–response study.^[25]^ Because dexmedetomidine has a more pronounced effect in unmyelinated C fibers than in Aα fibers in terms of blocking the hyperpolarization-activated current for hyperpolarization of the nerve,^[28]^ the DOM was relatively less affected by increasing doses.

The pain score results were similar to those for DOA. The NRS scores were lower in dexmedetomidine-treated patients than in those treated with normal saline at 12 h after ISB. In particular, the NRS scores were lower at 18 h after ISB in patients treated with 2 μg/kg of dexmedetomidine than in those treated with the other dexmedetomidine doses. However, despite the DOA in the 2 μg/kg dexmedetomidine group being the longest, there were no differences in the NRS scores at 24 and 36 h after ISB or in consumption of rescue analgesics between the groups. Previous studies have also indicated that pain scores were similar in patients treated with perineural dexmedetomidine to those in patients who received placebo after waning of the ISB effect.^[13,29]^ Fritsch et al compared the effect of dexmedetomidine 150 μg with that of placebo in ISB and found no difference in pain scores or in consumption of analgesics 24 h after ISB.^[13]^ In a study that compared the effects of 0.5 μg/kg IV or perineural dexmedetomidine with those of placebo in ISB, the 24-h cumulative postoperative morphine consumption decreased in the dexmedetomidine groups but the pain scores at rest were not significantly different between the 3 groups 24 h after ISB.^[29]^ Therefore, although perineural dexmedetomidine extended the DOA of ISB, multimodal analgesia is still required during transition from active block to postoperative pain. Because the difference in DOS and DOA is about 40 to 70 min in dexmedetomidine-treated patients, it may be helpful to administer rescue analgesics or a loading bolus of PCA approximately 30 min after the numbness wears off.

The doses of perineural dexmedetomidine need to be selected carefully to achieve a balance between prolongation of analgesia and side effects. Although dexmedetomidine is administered perineurally, it can be absorbed and redistributed, so may exert effects that are mediated systematically, such as bradycardia and hypotension. In our study, patients who received perineural dexmedetomidine showed lower BP and HRs intraoperatively and during recovery, but the degrees of hypotension and bradycardia were not severe enough to cause substantial adverse effects. The hemodynamic effects of perineural dexmedetomidine have been variable in similar studies, but most have reported reversible lowering of BP and HR.^[10,13]^ Gillespie et al suggested that patients can tolerate a 30% to 40% decrease in mean arterial pressure safely during shoulder arthroscopy and that the hypotension induced may have the benefit of allowing better visualization and decreasing blood loss.^[30]^ Although our hemodynamic data do not confirm the safety of perineural dexmedetomidine in patients with cardiovascular compromise, we could conclude that a 2 μg/kg dose can be used without close monitoring or management of BP after anesthesia in tolerable patients. Interestingly, the systolic BP in the dexmedetomidine groups was higher than that in the control group 5 min after ISB. A possible mechanism for this finding may involve the initial vasoconstrictive effects of dexmedetomidine that occur via stimulation of peripheral α_2_ receptors.^[31]^ When dexmedetomidine is administered intravenously, arterial BP is increased initially by this mechanism.^[31]^ Intramuscular dexmedetomidine also showed a similar biphasic change in HR and mean arterial pressure in another study.^[27]^

Increasing doses of perineural dexmedetomidine was also associated with higher sedation levels in the volunteer study mentioned earlier.^[25]^ In our study, there were no statistically significant differences in sedation scores between any of the groups, and all patients were alert within 6 h after ISB. Because the average operating time was 80 to 100 min, the plasma dexmedetomidine concentration may have decreased to arousable level after emergence from general anesthesia. A plasma dexmedetomidine concentration of about 0.2 to 0.3 ng/mL allows rousable sedation,^[32]^ and 1 study showed that 150 μg of perineural dexmedetomidine resulted in a plasma dexmedetomidine concentration of 0.37 ng/mL 90 min after administration.^[13]^

Brachial plexus block can cause dyspnea because of phrenic nerve palsy. Phrenic nerve palsy is caused by spreading of local anesthetic and its duration is determined by the duration of the local anesthetic effect.^[33]^ Liu et al^[34]^ showed that clinically significant palsy may occur in up to 10% of the patients who undergo ultrasound-guided ISB. In our study, 9 (9.27%) of 97 patients complained of dyspnea, which is similar to the incidence of phrenic nerve palsy reported by Liu et al. Most patients had mild and transient symptoms that resolved spontaneously without any invasive intervention; however, 1 patient who received 1.5 μg/kg of perineural dexmedetomidine complained of dyspnea until 18 h after ISB. This finding may be attributable to prolonged phrenic nerve palsy. Hypoxemia may be caused by sedation because of systemic absorption of dexmedetomidine or dyspnea. In the present study, hypoxemia occurred only in dexmedetomidine-treated patients and the mean SpO_2_ in the dexmedetomidine groups was lower than that in the control group in the recovery room. However, the incidence was low and no patient required noninvasive or invasive ventilatory support and all patients recovered within 6 h with oxygenation via a face mask. Although we could not conclude that perineural dexmedetomidine worsens dyspnea or hypoxemia, careful vigilance is needed when performing ISB with perineural dexmedetomidine in high-risk patients.

Our study has some limitations. First, we did not measure the time to onset of sensory or motor block. Because the ISB was for postoperative analgesia and all patients underwent general anesthesia after ISB, we did not make much account of measuring the timing of onset of the block. Second, the patients’ weights and the proportions of ASA physical status II were higher in the groups that received dexmedetomidine 1 or 2 μg/kg than those in the control group. However, we administered the dose of study drug depending on the patients’ IBW and excluded the patients with underlying disease which can affect the results of the study. Third, we only assessed pain at rest and not during activity. The reason for this was that after arthroscopic shoulder surgery all patients wear abduction braces to fix the shoulder, and we expected that this would confound the pain assessment with activity. Finally, we did not evaluate the effects of a dexmedetomidine dose above 2 μg/kg, because we judged that a higher dose of dexmedetomidine may cause more systemic side effects. Therefore, it is not possible to state that 2 μg/kg is the maximum possible dose, and further research would be needed to assess the safety and efficacy of perineural dexmedetomidine at doses higher than 2 μg/kg.

## 5 Conclusion

The optimal dose of perineural dexmedetomidine in ultrasound-guided ISB for arthroscopic shoulder surgery was 2 μg/kg, and could be used for adequate prolongation of analgesic duration as about 20 h.

## Acknowledgment

The authors thank Ms Jung-Woo Lee for her statistical analysis of the study data.

## Author contributions

**Conceptualization:** Kwon Hui Seo, Jae Hyuk Kang.

**Data curation:** Hong Soo Jung, Jae Hyuk Kang, Jin-Young Jeong, Na-Re Han.

**Investigation:** Jae Hyuk Kang, Jin-Young Jeong, Na-Re Han.

**Writing – original draft:** Hong Soo Jung.

**Writing – review & editing:** Kwon Hui Seo, Yong-Shin Kim.
